# Paediatric Rheumatology Fails to Meet Current Benchmarks, a Call for Health Equity for Children Living with Juvenile Idiopathic Arthritis, Using Digital Health Technologies

**DOI:** 10.1007/s11926-024-01145-w

**Published:** 2024-03-11

**Authors:** Sonia Butler, Dean Sculley, Derek Santos, Xavier Girones, Davinder Singh-Grewal, Andrea Coda

**Affiliations:** 1 School of Bioscience and Pharmacy, University of Newcastle, 10 Chittaway Rd, Ourimbah, NSW 2258 Australia; 2 School of Health Sciences, Queen Margaret University, Musselburgh, EH21 6UU UK; 3 Faculty of Health Sciences, Universities de Catalunya, Via Laietana, 2. Planta 4, 08003 Barcelona, Spain; 4 Department of Rheumatology, Sydney Children’s Hospitals Network, Randwick and Westmead, Westmead, NSW 2145 Australia; 5 John Hunter Children’s Hospital, New Lambton Heights, NSW 2305 Australia; 6 Discipline of Child and Adolescent Health, University of Sydney, Camperdown, NSW 2006 Australia; 7 School of Women’s and Children’s Health, University of NSW, Sydney, NSW 2052 Australia; 8 School of Health Sciences, University of Newcastle, Chittaway Rd, Ourimbah, NSW 2258 Australia; 9 ‘Equity in Health and Wellbeing Research Program’ at the Hunter Medical Research Institute (HMRI), Lot 1, Kookaburra Circuit, New Lambton Heights, NSW 2305 Australia

**Keywords:** Paediatric, Juvenile idiopathic arthritis, Integrated care, Digital health, History

## Abstract

**Purpose of Review:**

This critical review begins by presenting the history of Juvenile Idiopathic Arthritis (JIA) management. To move the conversation forward in addressing the current shortcomings that exist in the clinical management of children living with JIA, we argue that to date, the advancement of successful treatments for JIA has been historically slow. Factors implicated in this situation include a lack of rigorous research, JIA being considered a rare disease, and JIA’s idiopathic and complex pathophysiology.

**Recent Findings:**

Despite the well-intended legislative changes to increase paediatric research, and the major advancements seen in molecular medicine over the last 30 years, globally, paediatric rheumatology services are still failing to meet the current benchmarks of best practice. Provoking questions on how the longstanding health care disparities of poor access and delayed treatment for children living with JIA can be improved, to improve healthcare outcomes.

**Summary:**

Globally, paediatric rheumatology services are failing to meet the current benchmarks of best practice. Raising awareness of the barriers hindering JIA management is the first step in reducing the current health inequalities experienced by children living with JIA. Action must be taken now, to train and well-equip the paediatric rheumatology interdisciplinary workforce. We propose, a resource-efficient way to improve the quality of care provided could be achieved by embedding digital health into clinical practice, to create an integrative care model between the children, general practice and the paediatric rheumatology team. To improve fragmented service delivery and the coordination of interdisciplinary care, across the healthcare system.

## Background

### Evolution of Juvenile Idiopathic Management

#### Paradox of the Paediatric Orphan

Pharmaceutical companies have, for a long time, shunned paediatric research by ignoring the importance of therapeutic trials of medication in children [1–4]. This was because pharmaceutical companies considered the paediatric population as a small and unlucrative market to sponsor, where their cost of rigorous clinical trials could not be reclaimed [5, 6]. There were also ethical issues related to the recruitment of children [1, 7], paediatric dosing, and the stress children experience with blood sampling [2].

Instead, pharmacological testing disproportionally represented adults [1, 4, 8]. In order to evade paediatric endorsement, pharmaceutical companies placed a clause on medication labels, not recommending their medications for paediatric usage [7, 9]. In fact, in 1991 in the USA, 81% of medications contained this disclaimer [10], and as a consequence, most medications prescribed for children were officially not licensed (unlicensed); or used outside of the terms of the license (off-labelled) [2–4]. This meant prescribers were left to draw on the results of adult studies to conclude the child’s therapeutic response and dosage requirements [1, 4], whilst making assumptions as to the physical and physiological differences between adults and children [7, 10], and the pharmacokinetic variances seen from childhood to young adulthood (neonates, infants, children, and adolescents) that can alter drug absorption, distribution, metabolism and excretion [4, 11]. As a result, most medications (67 to 91%) prescribed to children had no efficacy, safety or quality testing [2, 4, 12], potentially exposing children to ineffective treatments, medication toxicity or other serious adverse events [13, 14].

#### Legislative Changes

Fortunately, over the last two decades, legislative changes came into place, boosting paediatric drug trials [7], and improving paediatric labelling [4, 15]. In the USA for example, the *Best Pharmaceuticals for Children Act* (2002) identified a priority list of medications that needed further study [7]. Whilst the *Pediatric Research Equity Act* (2003) required all new medications to include a paediatric assessment, unless waived [7, 15], within 5 years, information on efficacy and safety had been added to more than 400 medications, for neonates, infants, children, and adolescents [7, 15]; and these changes were often surprising. For Juvenile Idiopathic Arthritis (JIA), this brought about an increase in the dosage requirements (per-kilogram) for the commonly prescribed nonsteroidal anti-inflammatory drug (NSAID) Etodolac (now discontinued). Children aged 6 to 16 years required twice the lower dose recommended for adults [8]. Similar changes spread, for example in Australia (2014), the *Therapeutics Goods Administrator* adopted the *European Medicine Agency Guidelines on Pharmaceutical Development,* to ensure safe data generation and quality paediatric medications [16].

#### Rare and Ignored

Another unprofitable market ignored by pharmaceutical companies was the treatments for rare diseases [5]. In response, in the USA, the *Orphan Drug Act* (1983) was passed, aiming to stimulate investments by providing financial incentives to reduce research and marketing costs. Similar policies have been followed in Japan (1993), Australia (1997) and Europe (1999) [17], to promote pharmaceutical development in small populations [18, 19]. For JIA, these legislative changes improved the availability of treatments targeting specific subtypes [20].

#### Treatment Guided by Expert Opinion and Close Monitoring

Due to a lack of paediatric specific research, the treatment of JIA was conservatively based on adult rheumatic disease [21]. Adult mainstay treatment followed the principles of the *therapeutic pyramid* [22], in other words, an escalation of steps [23], to target excessive inflammation and pain [3, 21]. Generally, this was achieved through mild analgesia, NSAIDs such as salicylic acid (Aspirin), and if needed, corticosteroids [3, 21, 24]. Children were then closely monitored for side effects, such as gastrointestinal irritation and hepatotoxicity [24], osteopenia and growth retardation [25]. In addition, physiotherapy and splinting were keystone in preserving functional ability and preventing contracture [21]. Clinically, less than half of the children gained adequate control of their disease [26, 27], and remained at risk of chronic pain and disease-related disability [28]. Psychological support was also provided to the family by treating physicians, nurses, and if needed, a social worker [21].

#### Early Aggressive Treatment and the Promotion of Self-Management

Fortunately, in the 1980s, a major breakthrough occurred in rheumatology. A small window of opportunity to suppress the disease was identified in adult research studying early arthritis [29]. This turned the principles guiding the *therapeutic triangle* upside down [30], as the focus of care turned to early aggressive treatment to control disease and prevent tissue damage [23, 29]. The disease-modifying antirheumatic drugs (DMARD), methotrexate (MTX), became the forefront of treatment, for children that failed to respond sufficiently to the NSAIDs [3, 31]. MTX is a folic acid analogue that inhibits the folate pathway, interfering with tissue cell reproduction [32], transforming the outlook for many children with JIA [29]. The use of NSAIDs and corticosteroids moved to become an adjunctive treatment [28]. During this MTX era, a strong focus was also placed on educating children and their parents to promote medication adherence to optimise efficacy; and to monitor for side effects, because of the safety issues associated with MTX, for example increased liver enzymes [29].

#### A New Focus: The Pathogenesis of JIA

Through advancements in molecular medicine, a greater understanding was developed on inflammatory arthritides and cytokine networks [33]. This resulted in therapeutic treatments focusing on the pathogenesis of JIA, specifically the proinflammatory cytokines such as tumour necrosis factor alpha [TNFα], interleukin-1 [IL-1], and interleukin-6 [IL-6]; and the signalling mediators that regulated the B-cell and T-cell lymphocytes [34]. This major breakthrough is known as the biological era [35], increasing the number and types of biological DMARD treatments, that are based on varying modes of action, for example, Etanercept (TNFα antagonist) [32], Anakinra (IL-1 antagonist) and Tocilizumab (IL-6 inhibitor) to block the inflammatory process [34]. This brought about new treatments for individual subtypes.

Importantly, recently published cohort studies are now recording advancements in clinical care, post the biological era. Including, for many children, inactive disease (range 40–50%) [35], and disease remission (range 33 to 47%) [36••, 37•]; resulting in a marked reduction in disease damage [35]. However, there is still a substantial proportion of children whose course of disease could further be improved [35]. The need for close monitoring is also still needed, due to the antagonistic affects these medications can have on immunological pathways, placing children at an increased risk of infection [38].

#### Statement of Current Benchmark

The current benchmark for the management of JIA recommended by the *NSW Agency for Clinical Innovation* [39], the *British Society for Paediatric and Adolescent Rheumatology* [40], the *American College of Rheumatology* [41, 42], and the *Canadian Rheumatology Association* [43] encourages the following: early referral to health professionals specialising in paediatric rheumatic diseases, to support early diagnosis and the initiation of treatment. This treatment, then needs to be frequently assessed and adjusted accordingly, to achieve inactive disease and no pain [43].

#### Specialised Paediatric Rheumatology Centres

Further, to achieve the best possible health outcome, the management of JIA requires the support from a diverse interdisciplinary team, over a long period of time [44]. The forefront of this support is provided through specialised Paediatric Rheumatology (PR) Centres, typically based in tertiary children’s hospitals in major capital cities. These centres allow children and parents access to a *one stop shop* that delivers a range of holistic support and treatments that target both rheumatological and paediatric specific needs [39, 45, 46]. This is important because there are major differences between adult and paediatric care [39]. For example, physiotherapists may provide guidance on school physical education, sporting and home programs [46]. Nursing may recommend supportive strategies to parents for their child and their siblings [40] or provide practical advice on how to administer medications to children [40, 46], whilst podiatry monitors foot health and motor milestone development [47, 48], and ophthalmology screens eye health, in children too young to report symptoms [49•]. A crucially essential interdisciplinary care model needed to be available to all.

#### Global Challenges Hindering Paediatric Rheumatology

Internationally, the current demand on PR services far exceeds supply because of chronic shortages in the PR workforce [39, 50–52]. In fact, in Australia in 2021, there were only 20 PRs instead of the 61 recommended to meet the current workload. This means there is a 67% deficit, based on the minimum standards of care [53]. Globally, this workforce shortage shadows at 88%, with Asia and Africa being the worst affected locations [51, 54], despite their large paediatric populations [54]. Africa, for example, has only 10 PR centres across the 54 countries [55]; and China, 50 centres, even after three decades of pursuing improvements [56]. Also, in most countries, this workforce shortage is exacerbated in regional and rural areas [39]. Therefore, the standard of PR care falls below the expected benchmark [39], and this workforce shortage is expected to become worse [50], suggesting a serious problem and the delivery of suboptimal care [39, 57]. This will place children at risk of delayed and inadequate treatment, and poor outcomes from their disease.

One main reason has been the slow recognition of PR as a subspecialty [58–60]. As a result, medical schools have provided minimal paediatric education on musculoskeletal and rheumatic diseases [60], which has limited PR exposure and the uptake of residency programs offering PR rotations [61]. Consequently, this has had an impact on recruitment into the PR workforce, and a lack of trained staff to teach, and conduct research [52, 60, 62, 63]. This lack of knowledge then transcends across the disciplines, for example in orthopaedics and by general paediatricians [60, 64, 65]. It is not then surprising that those healthcare practitioners who are at the first point of contact with the healthcare system: general practitioners, emergency room physicians and school nurses, often overlook a child’s joint swelling and restriction [65, 66]. This results in diagnostic errors and delayed referral to the specialised PR centres, hindering a correct diagnosis and the provision of appropriate treatment [65, 67]. Concerningly, a cohort study of 130 PR centres across 49 countries, identified that these diagnostic delays were further intensified in low Gross Domestic Profit (GDP) countries [68••]. Inevitable, considering low GDP countries are still being inundated with communicable diseases, and their health care resources are limited. Further hindering their ability to apply the current standards of care [54, 69–71]; resulting in higher rates of disease activity, and more disease damage [68••].

Recently in Australia, the *National Action Plan for the Health of Children and Young People 2020–2030*, has put forth the importance of raising awareness of rare diseases, to enhance support, and improve health promotion, to allow children to thrive [72]. In addition, there is a call for new innovative approaches to address the current health inequalities, and indeed, better equipped the healthcare workforce in identifying and addressing a child’s healthcare needs [72]. Therefore, it is imperative that the way forward in JIA management is to understand the current barriers that need to be addressed in the management of JIA, to finally improve the health care disparities in access, treatment, and outcomes for children living with JIA.

We propose, a resource-efficient way to improve the level of care provided could be achieved by embedding digital health into clinical practice, through the use of digital sharing technologies [73]. For example, to create an integrative care model between the children, general practice and the PR team. To improve fragmented service delivery and the coordination of interdisciplinary care, across the healthcare system [74]. Also, essentially, allowing for ongoing monitoring and timely support to children with JIA, when needed.

#### Rise in Telehealth Consultations

Understandably, the COVID-19 pandemic generated a rise in telehealth in PR. Despite the criticism, because physical examinations are needed, Paediatric Rheumatologists confirm they were still able to verify diagnosis and treatment responses by using repeated visualization and feedback from parents [75]. However, several studies report, the assessments which commonly support treat-to-target decisions were not fully utilised, when compared to face-to-face consultations [76, 77]. Therefore, improvements are needed in the delivery of the virtual examination. Foremost, current assessment tools need to be validated [77], or adapted [78•], to ensure ease of use, and their virtual effectiveness [77]. As seen, for example, with the Video-pGALS (video paediatric Gait Arms Legs Spine) [78•], or eVAS (electronic visual analogue scale) [79].

#### New Emerging Models of Digital Healthcare

In the past decade, there have been profound changes in the types of digital technologies that have become available to support chronic disease management [80]. Mobile digital devices such as smartphones, tablets and smart watches can now connect to the Internet from almost any location [81, 82]; and are capable of reliably monitoring and assessing in real-time, crucial health indicators that could be used to support successful PR management.

For JIA, the benefits of remote monitoring are emerging [83]. For example, Stinson et al. reported that real-time pain assessments in electronic pain diaries, reduced recall bias [84]. Whilst Doeleman et al. demonstrated that quality of life questionnaires assessed through an app, asserted disease activity [85•]. Further, completing web-based assessments, before the Paediatric Rheumatologist consultation improved time efficiency [84] and promoted psychosocial conversation [86]. In addition, longitudinal monitoring of pain, medication adherence, and physical activity can be viewed on a web-based platform, captured through smart watch and mobile app technology [87]. However, most of this research is still at a very early stage of evaluation [83, 87]. Nonetheless, it is still laying the foundations to a new model of healthcare delivery. Notably, this model of digital health care also asserts the principles of person-centred care, because it requires engagement from children [84, 85•, 86, 87], and has the potential to improve communication between the child, parent and their wider healthcare team [88].

Therefore, it is crucial that future research and advancements in 5G digital health interventions should directly focus on addressing the long-standing health inequalities experienced by children with JIA, particularly for those living in remote or regional areas; in fact, any area or country far from Paediatric Rheumatologist and specialised PR centres.

## Conclusion

The significance of this critical review is to provide an overview of JIA management, by highlighting the journey of the paediatric orphan through to the current benchmark of best practice, in order to identify the long-standing limitations that have hindered the development of successful JIA management. In addition, a strong emphasis was placed on the need to frequently assess and modify JIA-related treatments accordingly, to achieve inactive disease and no pain. Yet meeting this current benchmark is difficult. The demand for services far exceeds service delivery, due to the global PR workforce shortage, resulting in, for many children, suboptimal care. Raising awareness of these issues is the first step in reducing these current health inequalities, to ensure health care organisations and policy makers understand the need, to increase, and better equip the PR interdisciplinary workforce through using digital health technology.

## Data Availability

The data that support the findings of this study is available on request from the corresponding author, sonia.butler@newcastle.edu.au.
